# Changes in interleukin-1 signal modulators induced by 3,4-methylenedioxymethamphetamine (MDMA): regulation by CB2 receptors and implications for neurotoxicity

**DOI:** 10.1186/1742-2094-8-53

**Published:** 2011-05-19

**Authors:** Elisa Torres, Maria D Gutierrez-Lopez, Andrea Mayado, Ana Rubio, Esther O'Shea, Maria I Colado

**Affiliations:** 1 Departamento de Farmacologia, Facultad de Medicina, Universidad Complutense, 28040 Madrid, Spain

## Abstract

**Background:**

3,4-Methylenedioxymethamphetamine (MDMA) produces a neuroinflammatory reaction in rat brain characterized by an increase in interleukin-1 beta (IL-1β) and microglial activation. The CB2 receptor agonist JWH-015 reduces both these changes and partially protects against MDMA-induced neurotoxicity. We have examined MDMA-induced changes in IL-1 receptor antagonist (IL-1ra) levels and IL-1 receptor type I (IL-1RI) expression and the effects of JWH-015. The cellular location of IL-1β and IL-1RI was also examined. MDMA-treated animals were given the soluble form of IL-1RI (sIL-1RI) and neurotoxic effects examined.

**Methods:**

Dark Agouti rats received MDMA (12.5 mg/kg, i.p.) and levels of IL-1ra and expression of IL-1RI measured 1 h, 3 h or 6 h later. JWH-015 (2.4 mg/kg, i.p.) was injected 48 h, 24 h and 0.5 h before MDMA and IL-1ra and IL-1RI measured. For localization studies, animals were sacrificed 1 h or 3 h following MDMA and stained for IL-1β or IL-1RI in combination with neuronal and microglial markers. sIL-1RI (3 μg/animal; i.c.v.) was administered 5 min before MDMA and 3 h later. 5-HT transporter density was determined 7 days after MDMA injection.

**Results:**

MDMA produced an increase in IL-ra levels and a decrease in IL-1RI expression in hypothalamus which was prevented by CB2 receptor activation. IL-1RI expression was localized on neuronal cell bodies while IL-1β expression was observed in microglial cells following MDMA. sIL-1RI potentiated MDMA-induced neurotoxicity. MDMA also increased IgG immunostaining indicating that blood brain-barrier permeability was compromised.

**Conclusions:**

In summary, MDMA produces changes in IL-1 signal modulators which are modified by CB2 receptor activation. These results indicate that IL-1β may play a partial role in MDMA-induced neurotoxicity.

## Background

Administration of the recreationally used drug 3,4-methylenedioxymethamphetamine (MDMA) induces a long-term toxicity of 5-HT nerve terminals in several areas of rat brain. The damage is reflected by a substantial decrease in the concentration of 5-HT and its metabolite, (5-HIAA), a reduction in the density of 5-HT uptake sites labelled with [^3^H]-paroxetine [1-4], a decrease in tryptophan hydroxylase activity [5] and a decrease in the immunoreactivity of fine 5-HT axons in cortex, striatum and hippocampus [6].

MDMA produces signs of neuroinflammation reflected as microglial activation and an increase in the release of interleukin-1β (IL-1β). Within 1-3 h following MDMA administration to rats there is an acute increase in IL-1β concentration in the hypothalamus and frontal cortex. The up-regulation of IL-1β levels appears at an early time-point after MDMA and is of short duration, levels returning to basal values 12 h after drug injection [7]. The IL-1β response is partially a consequence of MDMA hyperthermia and seems to be involved in the long-term 5-HT neurotoxicity since the i.c.v. injection of IL-1β in the rat brain enhances the long-lasting reduction in 5-HT transporters and 5-HT concentration induced by MDMA [8]. Nevertheless, the contribution of IL-1β to MDMA-induced neurotoxicity is not well established yet since IL-1β potentiates MDMA-induced damage at doses that exacerbates the immediate hyperthermic response induced by the drug. Therefore, actions of IL-1β on body temperature may contribute to the injury. In addition to increasing IL-1β, MDMA also increased the density of peripheral benzodiazepine receptor binding sites, labelled with [^3^H]-PK11195 ([(1-(2-chlorophenyl)-N-methyl-N-(1-methylpropyl)3-isoquinolinecarboxamide)], in hypothalamus and frontal cortex of the rat. This parameter could be reflecting an activation of microglia as revealed by subsequent immunohistochemical studies which show an increased in OX-42 immunoreactivity in both brain areas which is evident 3 h after drug injection and remains 24 h later [7].

Recent evidence indicates that MDMA increases the expression of cannabinoid CB2 receptors in frontal cortex and hypothalamus shortly after injection and that CB2 immunoreactivity co-localizes with the staining for the microglial cell marker OX-42, indicating the presence of CB2 receptors in microglial cells. Repeated administration of the CB2 agonist JWH-015 prevents the MDMA-induced microglial activation, reduces IL-1β release and provides partial protection against the serotonergic neurotoxicity induced by the drug [9]. Overexpression of the cannabinoid CB2 receptor in microglia during non-immune and immune pathological conditions is thought to be aimed at controlling the production of neurotoxic factors such as pro-inflammatory cytokines.

We have now examined the effect of JWH-015 on the changes induced by MDMA on endogenous IL-1 signal modulators in the frontal cortex and hypothalamus and explored the relevance of these actions on the protection exerted by this compound against MDMA neurotoxicity. The specific objectives of this study were as follows: 1) to determine the effects of MDMA on the levels of the IL-1 receptor antagonist (IL-1ra) and on the expression of the IL-1 receptor type I (IL-1RI) and evaluate the effect of JWH-015 on these changes; 2) to define the cellular location of IL-1β and IL-1RI by immunohistochemical staining; 3) to study the effect of i.c.v. administration of the soluble IL-1 receptor type I (sIL-1RI) on MDMA-induced hyperthermia and neurotoxicity; and 4) to evaluate the effect of MDMA on blood-brain barrier (BBB) permeability. There is evidence showing that BBB integrity is altered by a number of factors including increased levels of inflammatory cytokines [10] and free radicals [11-13], factors/mediators which are increased following MDMA injection [3,7].

## Methods

### Animals and drug administration

Male Dark Agouti rats (175-200 g, Harlan Laboratories Models, Barcelona) were used. MDMA induces a reproducible acute hyperthermic response in this strain [14,15] and also a reproducible long-term neurotoxic loss of 5-HT after a single dose [14]. Rats were housed in groups of 5 in conditions of constant temperature (21°C ± 2°C) and a 12 h light/dark cycle (lights on: 08 h 00 min) and given free access to food and water. MDMA was given at the dose of 12.5 mg/kg (i.p.), animals being sacrificed 1 h, 3 h, 6 h or 7 days after treatment. Room temperature at the time of MDMA administration was 21-22°C. The cannabinoid CB2 agonist, JWH-015 (2.4 mg/kg, i.p.), was given 48 h, 24 h and 0.5 h before MDMA. Doses and protocols of the cannabinoid agent used have been shown previously to be effective at modulating microglial functions [9,16,17].

MDMA (LIPOMED, Arlesheim, Suiza) was dissolved in saline (0.9% NaCl) and given in a volume of 1 mL/kg. Dose is reported in terms of the base.

JWH-015 was initially dissolved in DMSO (10 mg/mL), then diluted with 5% BSA in PBS and administered in a volume of 2 mL/kg.

Studies were performed in two brain areas, frontal cortex and hypothalamus since previous studies [7,8] showed that MDMA administration to Dark Agouti rats induces an increase in IL-1β levels in the hypothalamus and frontal cortex and that MDMA alters, in a region-specific manner, the mechanisms which regulate IL-1β production in the brain of Dark Agouti rats.

All experimental procedures were performed in accordance with the guidelines of the Animal Welfare Committee of the Universidad Complutense de Madrid (following European Council Directives 86/609/CEE and 2003/65/CE).

### Measurement of rectal temperature

Immediately before and up to 6 h after MDMA injection, temperature was measured by use of a digital readout thermocouple (BAT12 thermometer, Physitemp, NJ, USA) with a resolution of 0.1°C and accuracy of ± 0.1°C attached to a RET-2 Rodent Sensor which was inserted 2.5 cm into the rectum of the rat, the animal being lightly restrained by holding it in the hand. A steady readout was obtained within 10 s of probe insertion.

### IL-1ra immunoassays

Brain levels of IL-1ra were determined using a commercially available sandwich enzyme-linked immunosorbent assay (ELISA) system (human IL-1ra/IL-1F3, Quantikine^® ^Immunoassay, R&D Systems, Minneapolis, MN, USA). Samples were prepared by homogenization of frontal cortex and hypothalamus in 6 volumes of ice-cold buffer containing 50 mM Tris, 320 mM sucrose, 1 mM dithiothreitol, 10 μg/mL leupeptin, 10 μg/mL soybean trypsin, 2 μg/mL aprotinin and 0.2% phenantroline, pH 7.0. Samples were centrifuged at 14 000 × g for 10 min at 4°C. Protein was determined in the supernatant [18]. Samples were assayed in triplicate following the manufacturer's guidelines. The quantification of IL-1ra was performed using a standard curve of increasing concentrations of IL-1ra (31.2-2000 pg/mL). The optical density of each well was determined using a microplate reader (ELX808 IU, Ultra Microplate Reader, Bio-Tek Instruments, Inc) set to 450 nm (correction wavelength set at 540 nm). Intra-assay and inter-assay variations were less than 5% and 15%, respectively.

### Immunohistochemistry

Rats were anesthetized with sodium pentobarbital and perfused transcardially through the left ventricle with 200 mL of phosphate buffered saline (0.1 M PBS, pH 7.4) followed by 200 mL of 4% paraformaldehyde-PBS. Brains were removed, postfixed in the same solution for 4 h at room temperature and cryoprotected by immersion in 30% sucrose-PBS at 4°C. Brains were then frozen in powdered dry ice and stored at -80°C. The brains were sliced at 40 μm in the coronal plane through the whole frontal cortex and hypothalamus, and stored in cryoprotectant solution. Immunohistochemical studies were performed in hypothalamus and the sections were localized using a rat brain stereotaxic atlas [19].

For double labelling studies, cerebral free-floating sections were blocked by incubation with 0.5% BSA, 10% normal goat serum and 0.1% Triton X-100 for 30 min and incubated at 4°C with the appropriate primary antibodies (OX-42, Serotec, 1:400; anti-NeuN, Millipore, 1:100; anti rat-IL-1β, R&D Systems, 1:200; anti IL-1RI, Santa Cruz Biotechnology, 1:200) followed by the Alexa Fluor™ 488 donkey anti-mouse IgG (1:200), Alexa Fluor™ 594 donkey anti-rabbit IgG or Alexa Fluor™ 555 donkey anti-goat IgG (Invitrogen, Spain) secondary antibodies (1:500) and mounted in ProLong^®^Gold (Invitrogen). Images were acquired sequentially on a Leica TCS-SP2AOBS confocal microscope (Leica Microsystems, Heidelberg, Germany) for each fluorophore to avoid any cross-signal between them. Control experiments were performed in which sections were stained with each of the secondary antibodies or with the combination of them to rule out the possibility of reaction between them and images were taken using the same settings for each antibody staining.

IgG leakage from serum into the brain was assessed as a marker of vasculature damage. Rats were anesthetized with sodium pentobarbital and sacrificed. Brains were then frozen in powdered dry ice and stored at -80°C. The brains were sliced at 20 μm in the coronal plane through the hypothalamus. After three washes with 0.1 M PBS, sections were blocked by incubation with 0.5% BSA, 10% horse serum and 0.1% Triton X-100 for 60 min, incubated at 4°C overnight with the antibody Alexa FluorTM 488 donkey anti-rat IgG (1:500) and covered with ProLong^®^Gold (Invitrogen). Images were acquired as described above and ImageJ (NIH) software was used for image analysis. All images were converted to gray scale and blood vessels were outlined to provide an integrated gray scale value for statistical analysis.

### Western blot for IL-1RI immunoreactivity

Rats were killed by cervical dislocation and decapitation 1 h, 3 h or 6 h after MDMA administration, the brains rapidly removed and frontal cortex and hypothalamus dissected out on ice. Tissue was homogenized by sonication in ice-cold buffer containing 50 mM Tris, 320 mM sucrose and a number of protease inhibitors (Complete Mini, Roche, Madrid, Spain). Samples were centrifuged at 14 000 × *g *for 15 min at 4°C and protein determined in the supernatant [18].

The samples were boiled in laemmli buffer and proteins were resolved by 10% SDS-polyacrylamide gel electrophoresis (SDS-PAGE) and transferred to PVDF membranes. Nonspecific binding was blocked by incubation for 2 h in TBS buffer containing 5% skimmed milk. Membranes were incubated overnight at 4°C with the polyclonal anti IL-1RI as primary antibody (1:200) followed by incubation with goat anti-rabbit IgG-horseradish peroxidase (Amersham GE; 1:2000) for 2 h. Equal protein sample loading was confirmed by quantification of the β-actin signal.

### Quantification of IL-1RI immunoreactivity

Immunoreactivity was detected with an enhanced chemiluminescence (ECL) western blot detection system (Amersham Bioscience), followed by exposure to Amersham Hyperfilm ECL for 1-5 min. Different film exposure times were used to ensure that bands were not saturated. Quantification of specific bands on the film was performed using the Quantity One (USA) program. Each IL1-RI band density was normalised for protein content by referring it to its β-actin band density and then the IL1-RI expression in the different experimental conditions was expressed as a percentage of the control group.

### I.c.v. sIL-1RI administration

Rats were anesthetized with a mixture of isoflurane (3.5% for induction, 1-2% for maintenance; flow rate 1.5 L/min) and nitrous oxygen/oxygen mixture (30/70%) in air and placed in a stereotaxic frame secured in a Kopf stereotaxic frame with the tooth bar 3.3 mm below interaural zero. A 22 G guide cannula was implanted in the right lateral ventricle (according to the following coordinates: 7.9 mm rostral to the interaural line, 0.8 mm lateral to the midline and 3.1 mm from the skull surface; [19]). The cannula was secured to the skull as previously described [20]. I.c.v. injections took place 5 days after surgery through a 28 G injector (Plastics One, Roanoke, VA, USA) fitted in the guide cannula which protruded 1 mm beyond the guide. sIL-1RI (Sigma-Aldrich, Madrid, Spain) (3 μg/animal in 5 μl over 5 min) or PBS containing 0.1% BSA were administered i.c.v. 5 min before and 3 h after MDMA (12.5 mg/kg, i.p.) or saline (1 mL/kg, i.p.).

### Quantification of 5-HT transporter density by [^3^H]-paroxetine binding

[^3^H]-Paroxetine binding was measured in fresh hypothalamical and cortical tissue [2]. Briefly, tissue was homogenized in ice-cold Tris-HCl (50 mM; pH 7.4) containing NaCl (120 mM) and KCl (5 mM) using an Ultra-Turrax. The homogenate was centrifuged at 30 000 × g for 10 min at 4°C. The supernatant was discarded and the wash procedure repeated twice more. The pellet was finally resuspended in the Tris buffer at a concentration of 10 mg tissue/mL. Aliquots of tissue (800 μL) were incubated with [^3^H]-paroxetine (1 nM) for 90 min at room temperature in the absence and presence of 5-HT (100 μM) for determination of total and non-specific binding, respectively. Assays were terminated by rapid filtration through glass fibre filters and radioactivity determined by scintillation spectrometry. Protein was determined by the method of Lowry [21].

### Measurement of 5-HT concentration

Seven days after MDMA administration, rats were killed by cervical dislocation and decapitation, the brains rapidly removed and frontal cortex dissected out on ice. Tissue was homogenized and 5-HT measured by HPLC with electrochemical detection [3]. Briefly, the mobile phase consisted of KH_2_PO_4 _(0.05 M), octanesulfonic acid (0.16 mM), EDTA (0.1 mM) and methanol (16%), and was adjusted to pH 3 with phosphoric acid, filtered and degassed. The flow rate was 1 mL/min and the working electrode potential was set at +0.4 V.

The HPLC system consisted of a pump (Waters510) linked to an automatic sample injector (Loop 200 μL, Waters 717 plus Autosampler), a stainless steel reversed-phase column (Spherisorb ODS2, 5 μm, 150 × 4.6 mm) fitted with a precolumn, and a coulometric detector (Coulochem II, Esa, USA). The current produced was monitored by using an integration software package (Unipoint, Gilson).

### Statistics

Data from the ELISA, quantification of immunoreactivity and serotonergic parameters were analyzed using one-way ANOVA followed by the Newman-Keuls multiple-comparisons test when a significant F value was obtained. Statistical analyses of the temperature measurements were performed by two-way ANOVA with repeated measures using treatment as the between subjects factor and time as the repeated measure followed by Bonferroni as post test (GraphPad Prism 5).

## Results

### Effect of MDMA on brain IL-1ra levels

**Figure 1:**
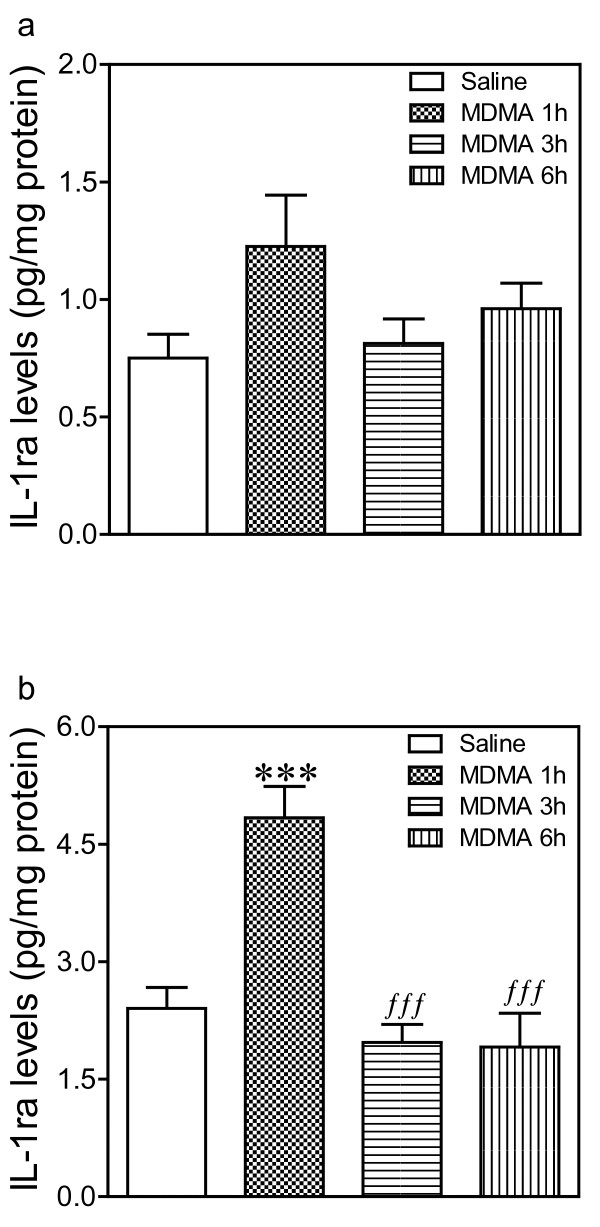
**Time-course of the changes induced by MDMA (12.5 mg/kg, i.p.) on IL-1ra (endogenous antagonist of IL-1 receptor) levels in a) frontal cortex and b) hypothalamus**. IL-1ra levels were measured 1 h, 3 h and 6 h after drug injection. Results shown as mean ± SEM (n = 6-8). Different from saline: ***p < 0.001. Different from MDMA-treated group at 1 h: ^*fff*^*p *< 0.001.

One-way ANOVA revealed a significant effect of treatment in hypothalamus (Figure 1b; F_3,20 _= 15.14, p < 0.001) but not in frontal cortex (Figure 1a; F_3,23 _= 2.28, p = 0.11, n.s.). Post-hoc analysis indicated that MDMA produced a substantial increase in IL-1ra levels in hypothalamus (101%, Figure 1b) 1 h after drug administration. This effect was not evident 3 h and 6 h later, times at which IL-1ra levels were similar to those found in the saline-treated group.

### Effect of MDMA on brain IL-1RI expression

**Figure 2:**
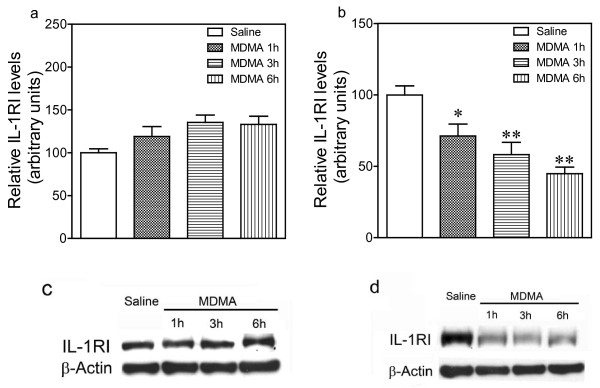
**Time-course of the changes induced by MDMA (12.5 mg/kg, i.p.) on IL-1RI expression in the frontal cortex (a) and hypothalamus (b)**. IL-1RI expression was measured 1 h, 3 h and 6 h after drug injection. Results shown as mean ± SEM (n = 5-13). Different from saline: *p < 0.05, **p < 0.01. A representative western blot shows immunoreactivity in frontal cortex (c) and hypothalamus (d).

One-way ANOVA revealed a significant effect of treatment in hypothalamus (Figure 2b; F_3,16 _= 7.28, p = 0.0041) but not in frontal cortex (Figure 2a; F_3,29 _= 2.55, p = 0.078, n.s.). Post-hoc analysis indicated that MDMA produced a substantial decrease in IL-1RI expression in hypothalamus 1 h (28.8%), 3 h (41.8%) and 6 h (55.1%) after administration with the greatest reduction occurring at 6 h (Figure 2b, d).

### IL-1RI expression in neuronal cells

**Figure 3:**
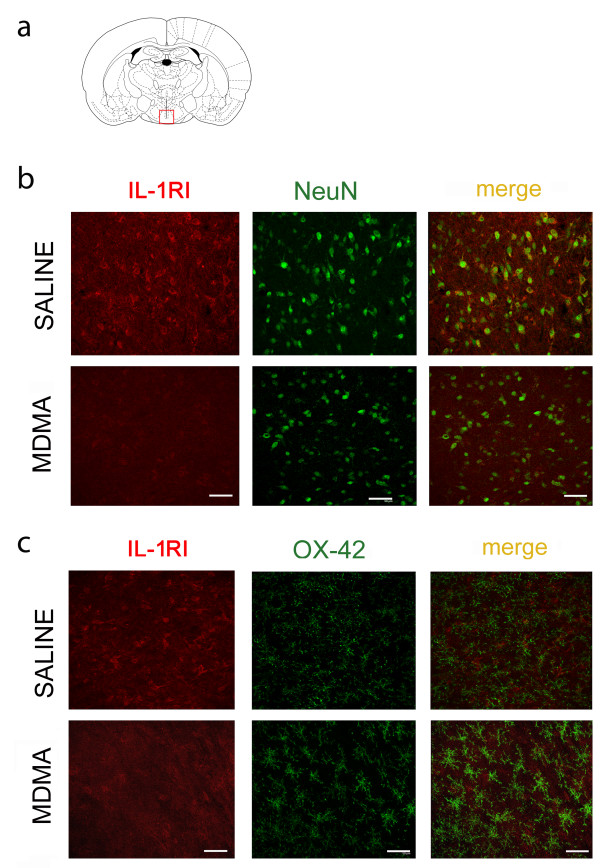
**Fluorescence images (40×) showing IL-1RI immunoreactivity (red staining) in hypothalamus of saline- and MDMA (12.5 mg/kg, i.p.)-treated rats (3 hours after injection)**. Double immunofluorescence with IL-1RI and NeuN (green staining, b: upper panel) or OX-42 (green staining, b: lower panel) revealed that IL-1RI is localized on neuronal cells, not on activated microglia. Scale bar = 50 μm. a: image showing area studied.

Saline-treated animals showed immunoreactivity for IL-1RI in the hypothalamus (Figure 3, red labelling). Double immunostaining studies using the NeuN antibody to label neuronal soma (Figure 3b, upper panel) and OX-42 to stain activated microglia (Figure 3c, upper panel), revealed that IL-1RI was localized in neuronal cells but not in microglia. In animals receiving MDMA, IL-1RI was also found in neurones 3 h after injection although the staining intensity was lower as expected by the decrease observed by western blot determinations (Figure 2d).

### IL-1β expression in neuronal and microglial cells

**Figure 4:**
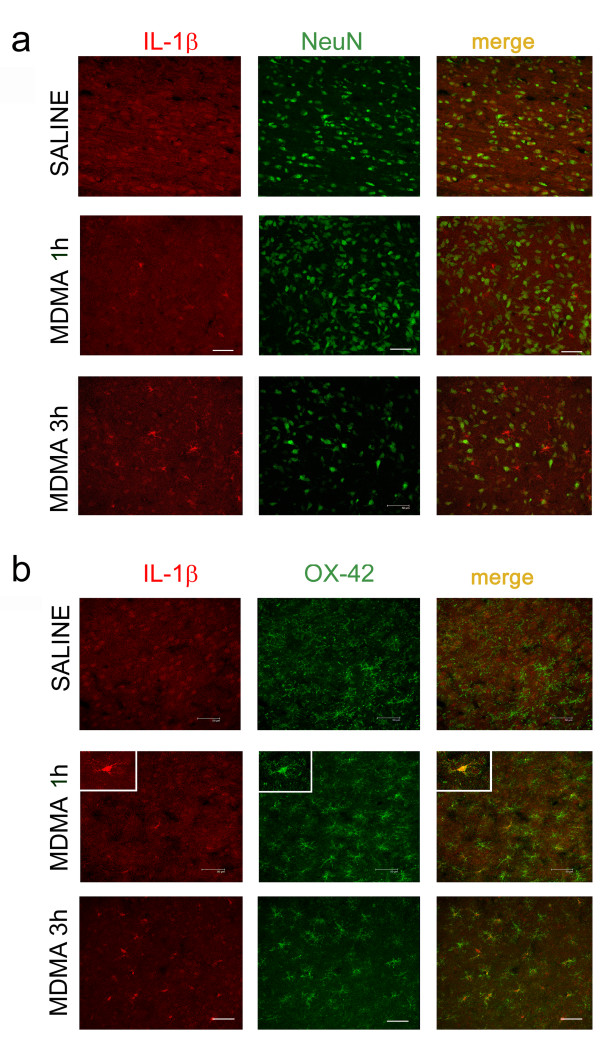
**Fluorescence images (40×) showing IL-1β immunoreactivity in the hypothalamus of saline- and MDMA (12.5 mg/kg, i.p.)-treated rats (1 h and 3 h after injection)**. Double immunofluorescence with IL-1β (red staining) and NeuN or OX-42 (both green staining, upper and lower panels respectively) revealed that IL-1β is constitutively expressed in neurons and that following MDMA the immunoreactivity for IL-1β overlaps with OX-42 immunostaining. Scale bar = 50 μm. Inset shows a stained microglial cell (63×). Same brain sections were studied as indicated in Figure 3a.

Saline-treated animals showed immunoreactivity for IL-1β in the hypothalamus (Figure 4, red labelling). Double immunostaining studies using the NeuN antibody to label neuronal soma (Figure 4a, upper panel) or the OX-42 marker for microglial cells (Figure 4b, upper panel) revealed that IL-1β was localized in neuronal cells in saline-treated animals. Following MDMA injection, IL-1β staining was also found in microglia, the immunoreactivity being greater 3 h after MDMA compared with that observed at the 1 h time-point.

### Effect of JWH-015 on MDMA-induced changes on IL-1ra release and IL-1R1 expression in hypothalamus

**Figure 5:**
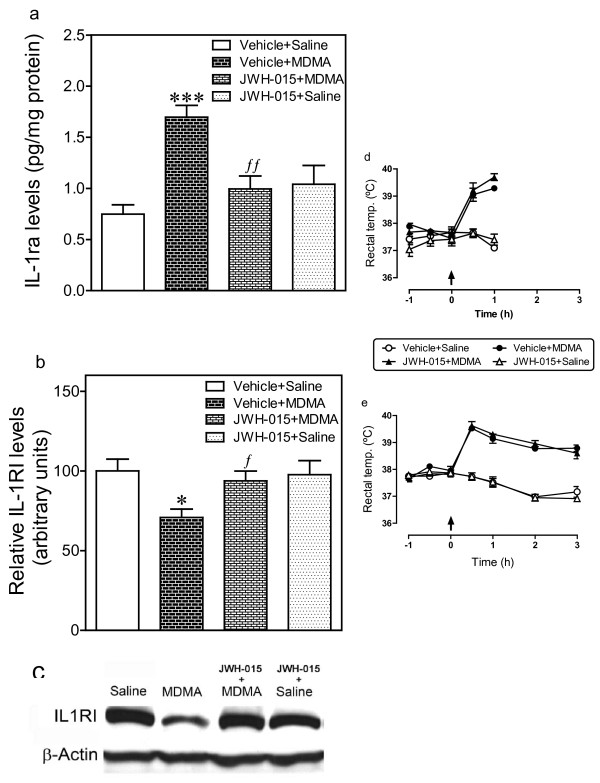
**Effect of JWH-015 on the MDMA-induced changes in IL-1ra levels (a) and IL-1RI expression (b) in hypothalamus. JWH-015 (2.4 mg/kg, i.p.) was injected 48 h, 24 h, and 0.5 h before MDMA (12.5 mg/kg, i.p.)**. For IL-1ra levels determination rats were killed 1 h after MDMA administration (a). IL-1RI expression was measured 3 h after MDMA (b). The effect of JWH-015 on MDMA-induced hyperthermia is also shown (d,e). Results shown as mean ± SEM (n = 4-8). Different from vehicle + saline group: *p < 0.05, ***p < 0.001. Different from vehicle + MDMA-treated group: ^*f*^*p *< 0.05, ^*ff*^p < 0.01. In panels (d, e) different from vehicle + saline group: *p < 0.001. A representative western blot shows immunoreactivity in hypothalamus (c).

JWH-015 was injected 48 h, 24 h and 0.5 h before MDMA. With regard to IL-1ra levels, one-way ANOVA revealed a significant effect of treatment (Figure 5a; F_3,27 _= 13.95, *p *< 0.0001). Post-hoc analysis indicated that JWH-015 significantly reduced the increase in IL-1ra levels induced by MDMA (126.6%, Figure 5a) such that the increase in IL-1ra levels was 32.7%. JWH-015 did not alter IL-1ra levels in the brain of saline-treated rats.

IL-1RI expression was studied by western blot (Figure 5c). Statistical analysis by one-way ANOVA revealed a significant effect of treatment in hypothalamus (Figure 5b; F_3,25 _= 4.56, *p *= 0.013). Post-hoc analysis indicated that JWH-015 prevented the reduction in IL-1RI expression induced by MDMA (Figure 5b) such that the immunoreactivity of IL-1RI was similar to that observed in the vehicle-treated group. JWH-015 did not alter IL-1RI expression in the brain of saline-treated rats.

JWH-015 affected neither the hyperthermia of MDMA nor the rectal temperature of saline-treated animals (Figure 5d, e). Two-way ANOVA indicated that there was a significant effect of treatment (Figure 5e: F_3,120 _= 45,7, *p *< 0.0001; Figure 5d: F_3,154 _= 152,9, *p *< 0.0001), time (Figure 5e: F_4,120 _= 27,3, *p *< 0.0001; Figure 5d: F_6,154 _= 28,43, *p *< 0.0001) and interaction (Figure 5e: F_12,120 _= 10.0, *p *< 0.0001; Figure 5d: F_18,154 _= 18,9, *p *< 0.0001). Bonferroni post test revealed that MDMA produced a hyperthermic that was not modified by JWH-015.

### Effect of sIL-1RI on the MDMA-induced neurotoxicity

**Figure 6:**
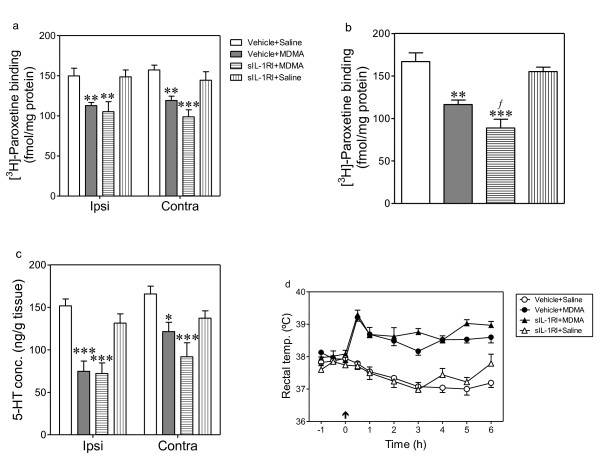
**Effect of intracerebroventricular injection of sIL-1RI on the MDMA-induced reduction of 5-HT transporters in frontal cortex and hypothalamus (a,b), cortical 5-HT concentration (c) and hyperthermia (d)**. sIL-1RI (3 μg) was administered 5 min before and 3 h after MDMA (12.5 mg/kg, i.p.), rats being killed 7 days later. Results shown as mean ± SEM (n = 5-9). Different from vehicle + saline group: *p < 0.05, ** *p *< 0.01, ***p < 0.001. Different from vehicle + MDMA-treated group: ^*f*^*p *< 0.05. In panel (d) different from vehicle + saline group: *p < 0.001.

Seven days after MDMA administration, the density of 5-HT transporters and the concentration of 5-HT was significantly reduced in the ipsi- and contralateral frontal cortex (Figure 6a,c). The density of 5-HT transporters was also reduced in hypothalamus (Figure 6b). Administration of sIL-1RI (3 μg, i.c.v.) significantly potentiated the reduction of 5-HT transporters induced by MDMA in the hypothalamus (Figure 6b) but it did not alter the 5-HT parameters in frontal cortex (Figure 6a,c). sIL-1RI did not modify the 5-HT levels (Figure 6a,c) nor density of 5-HT transporters (Figure 6a,b) in the brain of saline-treated rats.

### Effect of sIL-1RI on the MDMA-induced hyperthermia

Two-way ANOVA indicated that there was a significant effect of treatment (F_3,268 _= 128.2, *p *< 0.0001), time (F_9,268 _= 5.69, *p *< 0.0001) and interaction (F_27,268 _= 5.14, *p *< 0.0001). Bonferroni post test revealed that MDMA produced a hyperthermic response which peaked 30 min after treatment and was sustained for up to 6 h after treatment. sIL-1RI (3 μg, i.c.v.) affected neither the hyperthermia of MDMA nor the rectal temperature of saline-treated animals (Figure 6d).

### Effect of MDMA on BBB permeability

**Figure 7:**
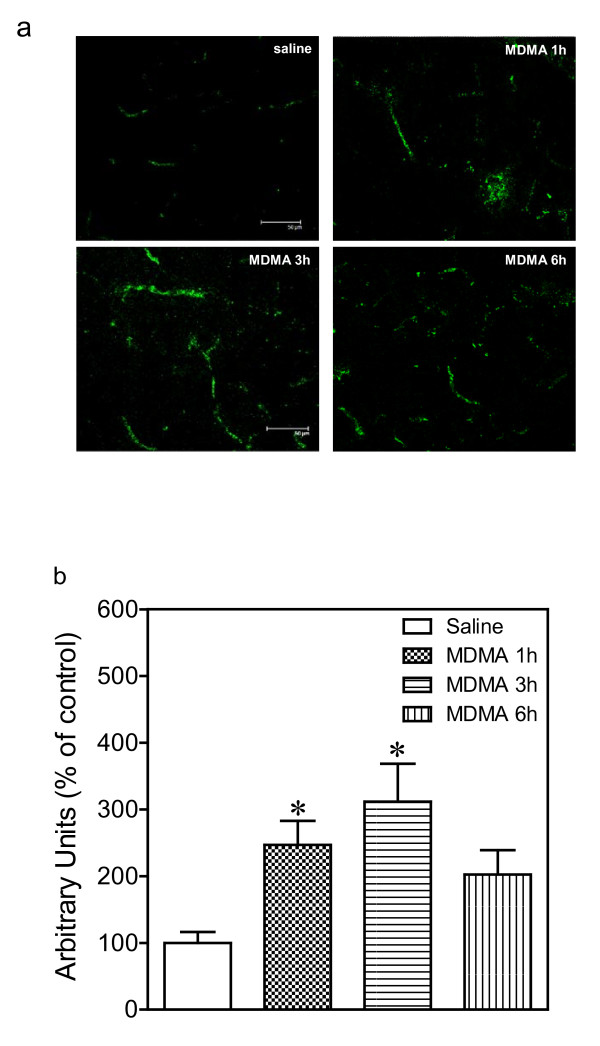
**Time-course of the changes induced by MDMA (12.5 mg/kg, i.p.) on IgG expression in hypothalamus**. Fluorecence images (40×) of representative IgG immunostained sections (a) of hypothalamus and quantification of intensity of immunostaining (b). Scale bar = 50 μm. Rats were killed 1 h, 3 h and 6 h after MDMA injection. Results shown as mean ± SEM (n = 4-6). Different from saline: *p < 0.05.

BBB permeability was visualized and quantified by means of IgG immunostaining (Figure 7a). One-way ANOVA revealed a significant effect of treatment in hypothalamus (Figure 7b; F_3,17 _= 5.122, *p *= 0.0113). Post-hoc analysis indicated that MDMA produced a substantial increase in hypothalamic IgG leakage (147%, Figure 7b) 1 h and (212%, Figure 7b) 3 h post-injection. This effect was not evident at the 6 h time point.

## Discussion

The current study demonstrates that MDMA produces differential regulation of several modulators of IL-1 signalling in hypothalamus and frontal cortex. Thus, in the hypothalamus, the drug induces a pronounced and transient increase in IL-1ra levels and a sustained marked reduction of IL-1RI density that lasted at least 6 h. Neither of these two changes is observed in frontal cortex. The differential effects induced by MDMA on IL-1β signal modulators could be a consequence of the intensity of action of MDMA on IL-1β release in these brain areas. Previous studies from our laboratory have shown that following MDMA, IL-1β levels are much higher in the hypothalamus than in frontal cortex [7]; the basal levels of IL-1β also being lower in frontal cortex.

The IL-1β pro-inflammatory effects are balanced by IL-1ra, a naturally occurring IL-1 receptor antagonist which is structurally related to IL-1β and binds to the type I IL-1 receptor (IL-1RI) competitively displacing IL-1β from the receptor complex without inducing any biological response [22]. In fact, there is an up-regulation of IL-1ra in various models of neurodegeneration [23-25] or after infectious stimuli [26]. In line with this, IL-1ra administration to rodents attenuates the neuroinflammatory response and neuronal damage mediated by IL-1β following several insults [27-32]. The greater IL-1β content in the hypothalamus compared with frontal cortex as early as 1 h after MDMA injection could trigger the release of IL-1ra in an attempt to limit the effects of the released IL-1β. In fact, much evidence indicates that the effectiveness of IL-1β signal transduction at the IL-1RI depends on the ratio between IL-1ra and IL-1β. In vivo studies reveal that relatively high IL-1ra to IL-1β molecular ratios are necessary for an effective blockade of IL-1β effects (at least 25:1, [33]). In our hands, in the hypothalamus, IL-1β levels increased 400% after MDMA dosing [7], while the increase in IL-1ra levels was 100% (this paper). This means that although an increase in IL-1ra levels may contribute to limiting the effects of IL-1β mediated through IL-1RI, the extent of this increase might not be sufficient to circumvent IL-1β signalling.

IL-1β signal transduction occurs via IL-1RI that is expressed at the cell surface; it binds IL-1β and leads to intracellular signalling by association with the IL-1 receptor accessory protein (IL-1RAcP). MDMA also produces a region-specific response in IL-1RI expression which may be a consequence of the different relative levels of IL-1β and IL-1ra in these brain areas. The more pronounced release of IL-1β in the hypothalamus after MDMA injection may lead to a down-regulation of IL-1RI that could be considered an additional defence mechanism to block the acute effects of IL-1β. Previous studies using radioligand binding in tissue homogenates or by autoradiography have reported a reduction in the neuronal density and affinity of IL-1 receptors after systemic administration of lipopolysaccharide at doses producing IL-1β release [34,35]. These findings indicate that increased levels of IL-1β regulate IL-1R1 receptor expression resulting in down-regulation.

In the current study we used immunohistochemistry and double-labelling immunofluorescence to investigate the cell types expressing IL-1RI. IL-1RI immunoreactivity was present in a constitutive way in the hypothalamus of saline-treated rats and double-staining revealed neuronal expression of IL-1RI since it shows receptor co-localization with NeuN. These findings support previous studies [36-39] and indicate that neurons are targeted by the endogenous IL-1β produced in the hypothalamus immediately after MDMA injection. The immunohistochemistry results show that staining for IL-1RI decreases following MDMA and reinforce those obtained by western blot. We could not find IL-1RI expression in microglial cells. These results are in agreement with those described previously [36,40] but are not, however consistent with those of previous reports [37,41,42].

Our results also show that IL-1β is constitutively expressed in hypothalamic neurons supporting previous studies showing that IL-1mRNA co-localize with large neuronal cell bodies [43]. There was no constitutive expression of IL-1β in microglial cells but it appeared in these cells coinciding with an increase in the number of OX-42 positive cells produced after MDMA administration. These data are in agreement with those showing that IL-1β is produced and released by microglia [36,44] during the early phase of activation and suggest that microglia is the main cell involved in propagating IL-1β effects in neuronal cells. In fact, we found that the intensity of IL-1β labelling in microglia was higher 3 h after MDMA compared with 1 h samples as expected according to the greater release of IL-1β based in our previous studies of time-course IL-1β levels after MDMA administration [7].

With respect to the role of IL-1β in the neurotoxicity induced by MDMA we have previously shown that i.c.v. administration of the cytokine potentiates the neurotoxicity produced by MDMA; however the exact role is difficult to assess since the IL-1β injection also increased the drug's hyperthermic response [8]. The data obtained following i.c.v. administration of sIL-1RI support at least a partial role for IL-1β in the neurotoxicity of MDMA in the hypothalamus. In addition to IL-1ra, IL-1β signalling is balanced by the soluble forms of its receptors which serve to capture the active molecules and thus reduce the levels available for signal transduction. IL-1β has very low affinity for IL-1sRI which, however, binds IL-1ra with high affinity [45,46]. Human sIL-1RI displays higher affinity for IL-1ra than membrane-bound IL-1RI, human sIL-1RI binds IL-1ra with a Kd of 14 pM, i.e. an affinity which is 200 times that of membrane-bound IL-1RI [47]. Both types of IL-1 inhibitor, i.e., sIL-1R and IL-1ra, may simultaneously be present at the inflammatory site and may interfere with each other's activity, one of which acts at the receptor level and the other at the ligand level thus leaving IL-1β activity unimpaired [46]. In fact, it has been reported that the inhibitory activity of IL-1ra is reduced by the simultaneous presence of sIL-1RI. Thus, in our study the binding of IL-1ra by sIL-1RI may be allowing the IL-1β-mediated effects.

Activation of the IL-1RI receptor by IL-1β may elicit a number of different events. Binding of the cytokine to the type I receptor allows the recruitment of IL-1RAcP, an action which is necessary for the initiation of the signalling cascade. Typically the resulting response involves the activation of NF-κB and mitogen-activated protein kinase pathways [48]. In fact, we have observed NF-κB activation following MDMA [49]. Activation of this transcription factor regulates the transcription of target genes that mediate the inflammatory response including IL-1β in a cyclic mechanism involving further IL-1β release and inflammation [50]. In addition, activation of NF-κB is associated with increased expression of several enzymes such as COX-2 and iNOS [51,52] which may in turn lead to the production of free radicals, elements which have been implicated both by us and by others in the 5-HT neurotoxicity produced by the drug [3,53-55]. Thus, activation of the IL-1RI receptor may ultimately lead to a potentiation of the neuroinflammatory response and an increase in oxidative stress.

Although these data point to a potential role for IL-1β in the neurotoxicity produced by MDMA, the studies with the CB2 agonist JWH-015 indicate that it cannot be the sole factor mediating damage. JWH-015 administration before MDMA prevented the increase in IL-1ra levels and the reduction of IL-1RI density in the hypothalamus. Previous studies showed that this compound also prevents the increase in IL-1β levels in hypothalamus [9]. Interestingly, the effects induced by JWH-015 on the MDMA-induced changes on IL-1 modulators are produced using a dosing regimen of JWH-015 that does not protect against MDMA-induced neurotoxicity [9]. It is worth noting that JWH-015 did protect against MDMA-induced neurotoxicity following regimens which in addition to inhibiting IL-1β produced inhibition of microglial activation. Thus, it appears that inhibition of microglial activation in addition to IL-1β production may play essential roles in MDMA neurotoxicity.

What can be stated unequivocally is that the ability of JWH-015 to prevent the MDMA-induced changes on IL-1 modulators is not related to an effect on body temperature. The hyperthermic response immediately following MDMA was not modified in rats also given the CB2 agonist (this paper; [9]).

What seems to be clear is that the changes induced by MDMA on IL-1β signal modulators are regulated by CB2 receptors. Following MDMA there is an increase in CB2 expression in activated microglia which is prevented in rats receiving concomitantly the CB2 agonist JWH-015 [9]. Activation of CB2 receptors might inhibit the conversion of microglia to a phenotype able to express and release IL-1β [9] and thus is probably responsible for the inhibition of IL-1β release from this cell type following MDMA. As a consequence of this, IL-1ra levels do not rise and IL-1RI are not down-regulated in MDMA-treated animals also given JWH-015.

There is evidence that expression of IL-1β in the CNS induces reversible breakdown of the BBB [56,57] and this effect has been linked to increased expression of matrix metalloproteinases (MMPs), a gene family of extracellular matrix enzymes which degrade junction proteins and change the permeability of the BBB [58]. The current study shows that MDMA increases IgG expression in the hypothalamus at 1 h and was maximal 3 h postinjection. BBB breakdown is often associated with increased extravasation of plasma proteins and high levels of immunoglobulin G (IgG) in brain, therefore, our results indicate that MDMA induces BBB disruption. The effect of MDMA on BBB integrity has not been studied in detail. To the best of our knowledge there is only one study examining the effects of acute MDMA on BBB dysfunction, brain edema, and cell injury in rats and mice [59]. This investigation shows the leakage of Evans blue dye, particularly in the cerebellum, hippocampus, cortex, thalamus, and hypothalamus shortly after MDMA and therefore, support the results reported in the current paper. Further studies are required in order to investigate possible mechanistic explanation.

## Conclusions

These findings indicate for the first time that MDMA induces changes in the upstream regulation of IL-1 signalling, producing an increase in IL-1ra and a decrease in IL-1RI. These changes are observed selectively in hypothalamus, but not in frontal cortex where the IL-1β release following drug injection is less pronounced. The changes are prevented by CB2 receptor activation. IL-1β is expressed in microglial cells shortly after MDMA while IL-1RI is expressed in the neuronal cell body of neurons suggesting that the release of IL-1β from microglia plays an important role in the neuroinflammatory response and that hypothalamic neurons are the main target cells for IL-1β. The fact that sIL-1RI administration potentiates the MDMA-induced loss of 5-HT transporters suggests a partial role for IL-1β signalling in MDMA-induced neurotoxicity.

## List of Abbreviations

5-HIAA: 5-hydroxyindoleacetic acid; 5-HT: 5-hydroxytryptamine; BBB: blood-brain barrier; BSA: bovine serum albumin; DMSO: dimethyl sulfoxide; IL-1β: interleukin-1beta; IL-1ra: IL-1 receptor antagonist; IL-1RI: IL-1 receptor type I; MDMA: 3,4-methylenedioxymethamphetamine; PBS: phosphate buffered saline; sIL-1RI: soluble IL-1 receptor type I.

## Competing interests

The authors declare that they have no competing interests.

## Authors' contributions

ET carried out ELISA and western blot assays and participated in immunohistochemical studies and design of the study. MDGL carried out immunohistochemistry, confocal microscopy and participated in the interpretation of the data. AM participated in western blot analysis and neurotoxicity study. AR carried out the study on IgG extravasation. EOS participated in the interpretation of data and helped to draft the manuscript. MIC participated in the design of the study, data analysis and drafting and revising the manuscript. All authors read and approved the final manuscript.
